# Response to “Limited capacity to retain phosphorus in the Baltic proper offshore sediments” by Karlsson and Malmaeus

**DOI:** 10.1007/s13280-018-1037-8

**Published:** 2018-02-26

**Authors:** Anders Stigebrandt

**Affiliations:** Department of Marine Sciences, University of Gothenburg, Box 461, 40530 Gothenburg, Sweden

Comment to: Karlsson, O.M, and J.M. Malmaeus. 2018. Limited capacity to retain phosphorus in the Baltic proper offshore sediments. *Ambio*. 10.1007/s13280-018-1019-x.

According to the time-dependent phosphorus budget model in Stigebrandt (2018), the winter surface water phosphorus (P) concentration c1 in the Baltic proper (BP) adjusts to the Total P Source (TPS). The model shows that the BP can be restored by turning off the Internal P Source (IPS) emanating from anoxic bottoms. At present, IPS accounts for about 70% of TPS. IPS may be turned off by (natural or man-made) sustained oxygenation of the deep bottoms. Using numerical values from Stigebrandt (2018), TPS would decrease from about 140 000 to about 42 000 tonnes year^−1^ if IPS is turned off. Figure 6 in Stigebrandt (2018) shows that c1 decreases by about 70% during the restoration process which means that the Total sink (Totsink), which is proportional to c1, also decreases by about 70%. The Internal P sink (Intsink), which accounts for about 84% of Totsink, would consequently decrease from about 118 000 to 35 000 tonnes year^−1^.

Intsink can be estimated as follows. A published estimate of P burial in oxic bottoms in the BP was unfortunately not found. However, the annual P sink in the (oxic) bottoms in the inner Stockholm archipelago (area 108 km^2^) equals 53 tonnes year^−1^, i.e. 0.49 tonnes km^−2^ year^−1^ (Karlsson et al. 2010). To obtain an estimate representative for the BP, I adjust for differing values of c1 in Stockholm Archipelago and the BP, 1.3 and 1.0 mmol m^−3^, respectively, by multiplying by 1/1.3 and get 0.38 tonnes km^−2^ year^−1^. Burial in the oxic bottoms of the BP (area about 170 000 km^2^) is thus estimated to about 65 000 tonnes year^−1^. Burial of P in organic matter in hypoxic and anoxic bottoms (area about 80 000 km^2^) is about 16 000 tonnes year^−1^ (0.2 tonnes km^−2^ year^−1^) (Jilbert et al. 2011). The export of P to the Bothnian Sea is about 15 000 tonnes year^−1^ (Savchuk 2005). The export of P by fish catch, about 1 100 000 tonnes year^−1^,^1^ can be estimated to about 5000 tonnes year^−1^ if the P content in fish equals 0.43% of the biomass (Hjerne and Hansson 2002). The exports of P by alga brought on land and by possibly increasing living biomasses in the BP are neglected. Intsink can thus be estimated to > 101 000 tonnes year^−1^. The BP thus seems to possess the internal sink capacity required by the model in Stigebrandt (2018).

The supposed dynamical effects of oxygenation of anoxic bottoms in BP are fundamentally different in the mathematical model in Stigebrandt (2018) and in the description provided by Karlsson and Malmaeus (2018). In Stigebrandt (2018), the sink processes adjust c1 to the new value of TPS when oxygenation turns off the IPS. The model explains the evolution of c1 in the BP from the 1950s to present time (Table 3 in Stigebrandt 2018). According to the description by Karlsson and Malmaeus (2018), oxygenation of the anoxic bottoms creates a new (single dose) P sink capacity of 100 000 tonnes (2 tonnes km^−2^) which reduces the P content in the water column by the same amount. Karlsson and Malmaeus (2018) turn down the existence of a sustained IPS. The P content in the water column of the BP increased by about 200 000 tonnes from 1980 to 2005 (e.g. Figure 2 in Stigebrandt 2018) despite the land-based P supply was almost halved during that period. It would be very interesting to hear Karlsson and Malmaeus quantitatively explain this without invoking a large sustained internal source.

## Footnotes

1 http://helcom.fi/action-areas/fisheries/commercial-fisheries/history-of-catches.

## References

[CR1] Hjerne O, Hansson S (2002). The role of fish and fisheries in Baltic Sea nutrient dynamics. Limnology and Oceanography.

[CR2] Jilbert T, Slomp CP, Gustafsson BG, Boer W (2011). Beyond the Fe-P-redox connection: preferential regeneration of phosphorus from organic matter as a key control on Baltic Sea nutrient cycles. Biogeosciences.

[CR3] Karlsson OM, Jonsson PO, Lindgren D, Malmaeus JM, Stehn A (2010). Indications of recovery from hypoxia in the inner Stockholm archipelago. Ambio.

[CR4] Karlsson OM, Malmaeus JM (2018). Limited capacity to retain phosphorus in the Baltic proper offshore sediments. Ambio.

[CR5] Savchuk OP (2005). Resolving the Baltic Sea into seven subbasins: N and P budgets for 1991-1999. Journal of Marine Systems.

[CR6] Stigebrandt A (2018). On the response of the Baltic proper to changes of the total phosphorus supply. Ambio.

